# Lipome du lobe profond de la glande parotide

**DOI:** 10.11604/pamj.2017.28.47.13000

**Published:** 2017-09-20

**Authors:** Hicham Attifi, Mehdi Lagtoubi

**Affiliations:** 1 Service d’Otorhinolaryngologie et Chirurgie Cervico-faciale, Hôpital Militaire Moulay Ismaïl, Meknès, Maroc

**Keywords:** Lipome, glande parotide, parotidectomie, Lipoma, parotid gland, parotidectomy

## Abstract

Lipomas of the parotid gland are benign tumors developing from the fatty tissue in the gland. They are rare, accounting for 0.6-4.4% of all benign tumors. Those located in the deep lobe of the gland are uncommon. Clinically, they are very difficult to diagnose. CT scan and especially MRI can support the diagnosis. Surgery is the treatment of choice but its modalities remain controversial. We report the case of a 52-year old female patient, with no particular past medical history, presenting with asymptomatic left preauricular mass evolving over 1 year and gradually increasing in volume. Clinical examination showed painless elastic swelling, measuring 2 × 1.5 × 1 cm, extending from the ear lobule to the left mandibular angle. Stensen duct wasn’t obstructed and saliva was clear. The patient showed no facial paralysis. CT scan showed perfectly delimited unilobular hypodense homogeneous mass in the deep lobe of the parotid gland. No suspected cervical lymphadenopathy was detected. MRI confirmed the presence of homogeneous, lipomatous intraparotid tissue process. After superficial parotidectomy and translocation of the branches of the facial nerve, a yellowish mass appeared under the buccal and mandibular branches of the facial nerve. The patient underwent complete resection up to the left parapharyngeal space. The postoperative course was uneventful. Anatomo-pathological examination confirmed the diagnosis of lipoma of the deep lobe of the parotid gland.

## Image en médecine

Les lipomes de la parotide sont des tumeurs bénignes se développant à partir du tissu graisseux de la glande. Ils sont rares représentant 0,6 à 4,4 % de toutes les tumeurs bénignes et ceux situés dans le lobe profond de la glande restent exceptionnels. Leur diagnostic clinique est très difficile à établir. La tomodensitométrie et surtout l'imagerie par résonance magnétique peuvent étayer le diagnostic. Le traitement est chirurgical mais ses modalités sont très controversées. Patiente âgée de 52 ans sans antécédents pathologiques particuliers a consulté pour une masse préauriculaire gauche asymptomatique évoluant depuis un an et augmentant progressivement de volume. L'examen clinique a retrouvé une tuméfaction élastique, indolore, de 2 × 1,5 × 1 cm, s'étendant du lobule de l'oreille à l'angle mandibulaire gauches. Le canal de Sténon était libre et la salive était claire. La patiente ne présentait aucun signe de paralysie faciale. La tomodensitométrie mettait en évidence une masse unilobulaire hypodense, homogène, parfaitement cloisonnée, située dans le lobe profond de la glande parotide. Aucune adénopathie cervicale suspecte n'était retrouvée. L'imagerie par résonance magnétique a confirmé la présence d'un processus tissulaire intraparotidien, homogène et de nature lipomateuse. Après la parotidectomie superficielle et réclinaison des branches du nerf facial, une masse jaunâtre apparaissait sous les rameaux buccal et mandibulaire du nerf facial. Son exérèse en bloc a été réalisée jusqu'à l'espace parapharyngé gauche. Les suites opératoires ont été simples. L'examen anatomopathologique a confirmé le diagnostic du lipome du lobe profond de la glande parotide.

**Figure 1:**
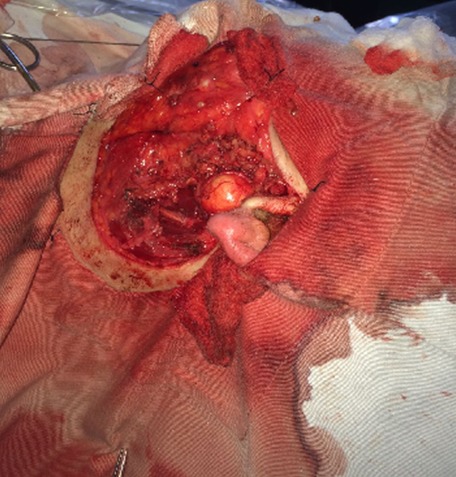
Vue peropératoire montrant le lipome au dépend du lobe profond de la parotide après parotidectomie superficielle

